# Impact of antibiotic treatments on the expression of the R plasmid *tra *genes and on the host innate immune activity during pRAS1 bearing *Aeromonas hydrophila *infection in zebrafish (*Danio rerio*)

**DOI:** 10.1186/1471-2180-12-37

**Published:** 2012-03-19

**Authors:** Leon Cantas, Paul J Midtlyng, Henning Sørum

**Affiliations:** 1 Department of Food Safety and Infection Biology, Norwegian School of Veterinary Science, Ullevålsveien 72, P.O. 8146, 0033 Oslo, Norway; 2 Norwegian Veterinary Alliance (veterinaryalliance.no), Reierstad, 2040 Kløfta, Norway; 3 Department of Basic Sciences and Aquamedicine, Norwegian School of Veterinary Science, Ullevålsveien 72, P.O. 8146, 0033 Oslo, Norway

## Abstract

**Background:**

The transfer of R plasmids between bacteria has been well studied under laboratory conditions and the transfer frequency has been found to vary between plasmids and under various physical conditions. For the first time, we here study the expression of the selected plasmid mobility genes *traD, virB11 *and *virD4 *in the 45 kb *IncU *plasmid, pRAS1, conferring resistance to tetracycline, trimethoprim and sulphonamide, using an *in vivo *zebrafish infection- treatment model.

**Results:**

Three days after oral infection of adult zebrafish with *Aeromonas hydrophila *harboring pRAS1, elevated expression of pro-inflammatory cytokine (TNF α, IL-1β and IL-8) and complement C3 genes in the intestine coincided with disease symptoms. Tetracycline, trimethoprim and an ineffective concentration of flumequine given 48 h prior to sampling, strongly increased expression of plasmid mobility genes, whereas an effective dosage of flumequine resulted in lower levels of mRNA copies of these genes relative to placebo treatment. Following effective treatment with flumequine, and ineffective treatments with a low concentration of flumequine, with trimethoprim or with sulphonamide, the intestinal expression of immune genes was strongly induced compared to placebo treated control fish.

**Conclusions:**

Treatment of zebrafish infected with an antibiotic resistant (Tc^R^, Tm^R^, Su^R^) *A. hydrophila *with ineffective concentrations of flumequine or the ineffective antimicrobials tetracycline and trimethoprim strongly induced expression of genes mediating conjugative transfer of the R-plasmid pRAS1. Simultaneously, there was a strong induction of selected inflammatory and immune response genes, which was again evident in fish subjected to ineffective treatment protocols. Our findings point to the essential role of therapeutic practices in escalation or control of antibiotic resistance transfer, and suggest that antibiotic substances, even in sub-inhibitory concentrations, may stimulate innate defenses against bacterial infections.

## Background

The zebrafish (*Danio rerio*) is a small tropical teleost that bridges the phylogenetic evolutionary gap between invertebrates and mammals in experimental biomedicine. It is evolutionarily closer to humans than fruit flies and nematodes, and is easier to work with and study than mice [1]. Recently, increased interest in using zebrafish for studies of human diseases as disparate as cancer, microbial infections and immune-pathological changes has evolved [2]. As an infection model, zebrafish have been employed for study of both human and fish pathogens [1,3-6].

*Aeromonas hydrophila *is a ubiquitous Gram-negative aquatic bacterium and opportunistic pathogen causing fatal hemorrhagic septicemia in several fish species including warm water and temperate aquaculture species [7-9]. In particular, *A. hydrophila *infections have been repeatedly reported from zebrafish facilities causing unusual [10] and sometimes high mortality rates [11]. Some strains of *A. hydrophila *have also been reported to be important human pathogens [12].

Conjugative R plasmids assigned to the *IncU *incompatibility group are widespread in environmental and fish pathogenic *Aeromonas *species worldwide [13]. An *IncU *representative, pRAS1, was detected in *Aeromonas salmonicida *from Norway [14]. This plasmid is very similar to an *IncU *plasmid derived from a human urinary tract pathogenic *Escherichia coli *in Eastern Germany as early as the 1970's [15]. The *IncU *pRAS1 has the potential to transfer between bacteria of diverse environmental compartments with high transfer frequency on solid surfaces [16].

The variable drug resistance region of *IncU *R-plasmids may contain a heterogenic collection of drug resistance genes and transfer systems that can mediate recombination and acquisition of additional resistance genes. In our study we used the 45 kb pRAS1 containing a class 1 integron, responsible for trimethoprim and sulfonamide resistance caused by *dfr16 *and *sul1*, respectively. In addition there is a Tn*1721 *transposon encoding tetracycline resistance by the Tet A determinant [14]. A highly conserved DNA backbone structure with a variable region encoding antibiotic resistance has been postulated for *IncU *group members [14]. The *IncU *plasmid pFBAOT6 (84.749 bp) was sequenced [17] and found to be almost identical with the *IncU *backbone of another plasmid RA3 (45.909 bp) [18]. Functional analysis of this broad-host-range *IncU *group of plasmids has demonstrated their self-transfer, replication and stable maintenance in alpha-, beta-, and gammaproteobacteria. The genetic functional transfer block of pRA3 consists of twenty-one different genes [18]. The mobility genes *traD, virB11 *and *virD4 *were selected from this functional block of the conjugative genetic system for analysis in this study.

The expression of a wide number of genes responsible for innate immune responses towards microbes in the intestine of adult zebrafish has been evaluated [19-23]. A recent study demonstrated the distribution of important innate antibacterial immunity mediators such as *peptidoglycan recognition protein *(*pglyrp*) and a factor that regulates neutrophilic cell densities and cytokines in the entire intestine of healthy zebrafish [24]. The bacterial pathogen recognition receptors (Toll-like receptors etc.) and signaling pathways activating the immune response (pro-inflammatory cytokines, hepicidin and heptoglobin etc.) are similar to those in mammals [25].

The aim of this study was, therefore, to assess the expression of transfer genes of pRAS1 caused by a pathogenic *A. hydrophila in vivo *in response to antibiotic treatments, while simultaneously monitoring selected inflammatory and innate immune system parameters.

## Methods

### Bacterial strains and growth conditions

*Aeromonas salmonicida *718 (NVI 2402/89) originally isolated from the head kidney of diseased Atlantic salmon in 1989, harboring a 25-MDa conjugative *IncU *plasmid, pRAS1, mediating resistance to oxytetracycline, trimethoprim and sulfadiazine was used as the donor strain. *A. hydrophila *strain (F315/10), originally isolated from a skin ulcer of freshwater reared salmon was used as the recipient strain, prior to zebrafish challenge. Both strains were cultured at 22°C on 5% cattle blood agar [blood agar base no 2, Difco] for 48 h (*A. salmonicida*) or 24 h (*A. hydrophila*).

### *In vitro *conjugation experiments

Conjugal transfer experiments were performed as described by Schmidt et al. [26]. In brief, donor *A. salmonicida *718 (carrying plasmid pRAS1) and recipient *A. hydrophila *F315/10 strains were grown overnight in Luria Broth (LB) with shaking at room temperature. Overnight cultures were diluted in LB to approximately 10^8 ^CFU/ml. Volumes of 100 μl of donor and recipient culture, respectively, were mixed and placed on the surface of a sterile 0.45 μm filter [Millipore] placed on the surface of an LB agar plate and incubated for 24 h at 22°C. The resultant colonies were suspended by vortexing the filter in 1 mL LB, pelleted and re-suspended in 100 μl of the same medium. Serial dilutions were then spread onto selective Luria agar (LA) plates supplemented with tetracycline (10 μg/ml), trimethoprim (10 μg/ml) and sulphonamide (200 μg/ml) for selection of trans-conjugants after 24 h incubation at 28°C. In parallel, the total number of recipients was estimated on LA after 24 h incubation at 28°C, a temperature not permissible for the donor strain. Conjugal transfer frequencies were calculated by dividing the number of trans-conjugants by the number of *A. hydrophila *recipients. The frequency of pRAS1 transfer was 1.8 × 10^-3^. Transfer of the R plasmid pRAS1 was confirmed by plasmid profile analyses and determination of the resistance pattern of the trans-conjugants as described by Cantas et al. [27].

### Plasmid isolation

The plasmids were isolated from trans-conjugants using a QIAprep Spin Miniprep kit [Qiagen, Hilden, Germany]. Plasmids were visualized under ultraviolet illumination following electrophoresis in 1% horizontal agarose gels and staining with ethidium bromide. Plasmid size was determined using BAC-Track supercoiled DNA markers [Epicentre].

### Zebrafish, challenge procedure and treatment

The zebrafish experiment was carried out at the experimental animal unit of the Norwegian School of Veterinary Science (NSVS), a facility licensed by the National Animal Research Committee. The experiment was approved by the same committee in accordance with national Regulations on Animal Experimentation. Adult zebrafish (> 6 months, TAB line) were supplied by the Aleström Zebrafish Lab (AZL), Oslo, Norway. The fish were fed commercial dry feed (SDS400, Special Diet Services, Witham, Essex, UK), twice daily according to AZL standard operational procedures. Water temperature was maintained at 22 ± 1°C throughout the experiment. Forty-two adult zebrafish of mixed gender (22 male, mean weight 441 mg/20 female, mean weight 514 mg) were allocated into 21 experimental units (sterile one-liter lab bottles: 2 fish per unit × 3 replicates × 7 experimental groups). All fish were starved for two days prior to experimental infection. The fish were anesthetized by immersion in benzocaine (ethyl p-aminobenzoate, 0.34 mg/ml) [Sigma-Aldrich]. Each fish was laid on its side on a moisturized paper tissue and a 20 μl saline suspension of pRAS1 bearing *A. hydrophila *F315/10 (1.6 × 10^8 ^CFU/ml) was administered into the stomach, using a micropipette fitted with a sterile feline urinary tract catheter (n = 18 units). The same volume of sterile physiological saline solution (0.9% NaCI) was employed for intubation of the uninfected control group (n = 3 units). The fish were immediately returned to the respective experimental unit and feeding resumed (every 12 h) to evaluate the appetite during the post challenge period. For the remaining part of the experiment, the fish were kept under continuous visual monitoring, with absence periods of less than 1 h. After 24 h the infected zebrafish were bath-treated with the following antibiotics [Sigma-Aldrich] added to the water: tetracycline (20 μg/ml), trimethoprim (20 μg/ml), sulphonamide (20 μg/ml) and subtherapeutic (0.06 μg/ml) or therapeutic (2 μg/ml) concentrations of flumequine, respectively. Distilled sterile water (1 ml/L) was used as a placebo treatment while the infection control groups were untreated.

### Sampling and culturing

To avoid mortality caused by the *A. hydrophila *infection prior to sampling, and to ensure maximum RNA preservation in bacteria sampled from the intestinal tract and in the intestinal tissue, fish from the challenged and control groups were observed every hour for three days following exposure. All fish were euthanized by decapitation at the end of the experiment. The abdominal cavity was opened by incision as described elsewhere Cantas et al. [28]. Entire intestinal samples were transversally sliced (< 0.5 cm) and immediately immersed in RNA*later *[Invitrogen] for bacterial and tissue RNA stabilization. Kidney samples from each sacrificed fish were examined bacteriologically for the presence of systemic infection. Specimens were streaked on 5% cattle blood agar and Brocalin agar [Merck, Darmstadt, Germany] as described by Cantas et al. [28].

### Gene expression

Total RNA from RNA*later*-stored tissue samples was extracted using Trizol Reagent [Invitrogen, Carlsbad, CA, USA]. Sterile 5 mm steel beads [Qiagen, Valencia, CA] were added for complete bacterial lyses in a Qiagen TissueLyser [Qiagen, Valencia, CA], run at 30 Hz for 5 min. Further processing was performed with the RNeasy kit [Qiagen, Valencia, CA]. Complete removal of DNA was achieved by treating the supernatant from the RNeasy processed samples with RNase-Free DNase Set [Qiagen, Valencia, CA]. Gel electrophoresis was used to confirm that isolated RNA was intact while the concentration and purity of the RNA were quantified using NanoDrop^® ^ND-1000 [NanoDrop Technologies, Delaware, USA].

Reverse transcription was performed with Superscript III Reverse Transcriptase [Invitrogen] following the manufacturer's instructions. cDNA amplifications were performed using previously published and novel designed specific primers [Table 1] by Primer 3 software [29]. Each primer (0.5 μl, 10 μM) was mixed with 18 μl of EXPRESS SYBR GreenER qPCR Supermix [Invitrogen]. Two μl template cDNA was used. Real-Time PCR tests were performed in duplicate with a Stratagene detection system [Stratagene, La Jolla, CA, USA] using optical grade 96-well plates, at the following conditions: 50°C for 2 min, 95°C for 2-10 min, 40 cycles of 95°C for 10-15 s and 55-60°C for 30-60 s. To determine the specificity of amplification, analysis of the product melting curve was performed after the last cycle of each amplification. Data was captured using Stratagene MxPro Mx3005P QPCR software.

**Table 1:** Primers employed for Real-Time PCR

Target gene	Sequence (5' to 3')		Reference or GenBank **accession no**.
*E. coli *16S rDNA	F	GCAGGCCTAACACATGCAAGTC	[30]
		
	R	TGCTGCCTCCCGTAGGAGT	

*traD*	F	ACGCCTCCTGTTCTGTTTCA	[DQ401103.1]
		
	R	ATCAGCCCGGTCAGATTGT	

*virB11*	F	GGATCAACTCAGCCACAAAAA	[DQ401103.1]
		
	R	CACCGTTCCGCTGTTCTATT	

*virD4*	F	GTTGTCCAGGGTAGCAGCAG	[DQ401103.1]
		
	R	TGGACAACCAGGAACAAGC	

*dfr16*	F	GACCTCATCCTCCGATGG	[AJ517790.2]
		
	R	TGGTCGGAGATATGGGTATAGAA	

*C3*	F	CGGACGCTGACATCTACCAA	[25]
		
	R	TCCAGGTCTGCTCTCCCAAG	

*IL-1β*	F	ATCAAACCCCAATCCACAGAGT	[25]
		
	R	GGCACTGAAGACACCACGTT	

*IL-8*	F	TGTTTTCCTGGCATTTCTGACC	[24]
		
	R	TTTACAGTGTGGGCTTGGAGGG	

*TNF α*	F	ACCAGGCCTTTTCTTCAGGT	[10]
		
	R	TGCCCAGTCTGTCTCCTTCT	

*ef1α*	F	TGCCTTCGTCCCAATTTCAG	[24]
		
	R	TACCCTCCTTGCGCTCAATC	

Amplification efficiencies were measured with the formula of E = 10^(-1/slope) ^by two-fold dilutions of cDNA as described by Bogerd et al. [31]. Expression of the plasmid target genes was normalized to *dfr*16, estimated to be the most stable endogenous reference gene on the plasmid for our *in vivo *experiment. The function describing the relationship between *C*_t _(threshold cycle) and *x *(log copy number) for *dfr*16 was: *C*_t = _-3.45*x *+ 13.98; *R*^*2 *^= 0.99. The comparative CT method [2^ΔCT ^method] was used to determine the expression level of analyzed genes [30]. The resultant fold units were calculated by dividing the normalized expression values with the placebo treated controls. Expression of the zebrafish inflammatory and immune response related target genes was normalized against expression of the housekeeping gene elongation factor 1 alpha *(ef1α) *[24] in challenged fish relative to sterile physiological saline solution intubated and placebo treated controls.

For absolute quantification of the total bacterial population of the gut, standard curves of 16S rDNA copy number were constructed using a PCR product of the 16S rRNA gene of *Escherichia coli*. The functions describing the relationship between *C*_t _(threshold cycle) and *x *(log copy number) for total bacteria was: *C*_t = _-3.19*x *+ 53.66; *R*^*2 *^= 0.99, as used by Castillo et al. [32].

To better address the activity of the innate immune response in zebrafish during the *A. hydrophila *infection, the transcription levels of the immune mediators: TNF α, IL-1β and IL-8 (pro-inflammatory cytokines) and C3 (complement system, acute phase protein) were evaluated. Fold changes in mRNA levels post-challenge and treatment were calculated in relation to the average mRNA levels of placebo treated fish.

### Statistical analysis

The effect of treatment on selected gene expression level was analyzed with Student's *t*-test as described by [33]. The results were expressed as mean ± SEM (standard error of the mean), based on variation between 6 adults per treatment group. Differences were considered significant at (*) *p *< 0.05, (**) *p *< 0.01 and (*** *p *< 0.001).

## Results

### Clinical symptoms and re-isolation of *A. hydrophila*

**Figure 1:**
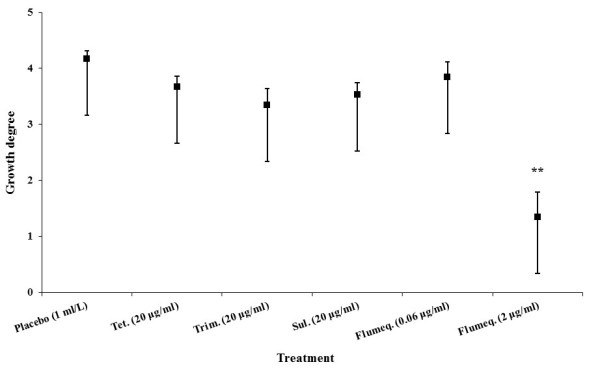
**Growth level median counts of *A. hydrophila *isolated from kidney samples of experimentally infected zebrafish, 48 h post antibiotic treatment (6 different treatment groups)**. Axis scale: absent = 0, very few = 1, few = 2, moderate = 3, rich = 4 and very rich = 5. Error bars represent ± SEM (6 adults per treatment group). Differences were considered significant at (**) *p *< 0.01 for total growth degree of placebo vs. other antibiotic treated zebrafish in each intestinal tissue analyzed.

No fish died within 3 days of the intubation challenge. All *A. hydrophila *inoculated zebrafish showed changes in external body color (pale, reddish coloration around gill covers), abnormal positioning in the aquarium (at the surface or near the bottom), increased gill ventilation frequency or lack of appetite within 24 h, while no such symptoms were seen in the uninfected control group. On termination of the experiment after 3 days, macroscopically visible ascites was observed in both the placebo treated fish and groups treated with ineffective antibiotics, whereas reduced clinical symptoms were noted in the group that had received effective treatment. Moderate to heavy growth of *A. hydrophila *in pure culture was detected from kidney samples of groups receiving placebo or ineffective treatments, whereas very low levels of *A. hydrophila *were isolated from groups of zebrafish exposed to effective antibiotic treatment [Figure 1].

### Immune response of zebrafish to *A. hydrophila*

**Figure 2:**
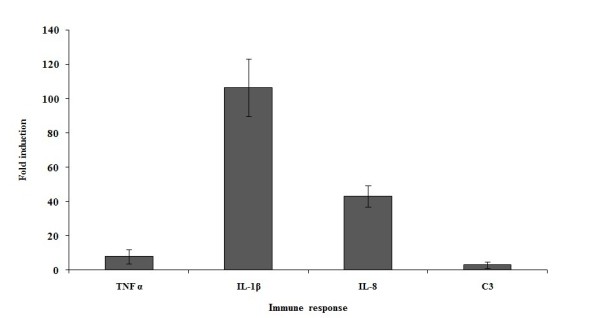
**Relative pro-inflammatory cytokine and complement C3 genes expression levels across the entire intestine of *A. hydrophila *infected and placebo treated adult zebrafish after harvesting 3 days post-challenge**. Expression levels are reported as fold change compared to average expression levels of uninfected (sterile physiological saline solution inoculated) control groups. Error bars represent ± SEM (based on variation between 6 adults per treatment group).

Compared to uninfected fish the transcription patterns of the innate immune response genes in placebo treated fish [Figure 2] were clearly raised and the transcription patterns of IL-1β (108 fold) and IL-8 (45 fold) genes were found to be substantially higher than TNF α (8 fold) and C3 (3 fold).

### Comparing the gut microbiota related 16S rRNA gene copy number under different antibiotic treatments

**Figure 3:**
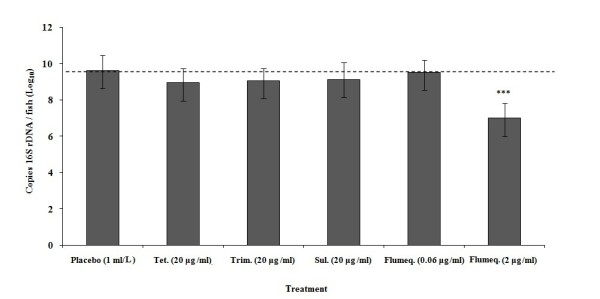
**Quantification of total gut microbiota in intestinal samples of experimentally infected and subsequently treated zebrafish based on 16S rDNA copy numbers**. Error bars reflect ± SEM (based on variation between 6 adults per treatment group). Differences were considered significant at (***) *p *< 0.001 for total 16S rDNA copy numbers of placebo vs. other antibiotic treated zebrafish in each intestinal tissue analyzed.

The copy numbers of 16S rRNA genes in the digestive tract significantly decreased following treatment with inhibitory doses of flumequine. The copy numbers obtained from ineffective antibiotic treatment groups were similar to those observed in the placebo treated group [Figure 3].

### Impact of antibiotic exposure on expression of the *tra *genes of pRAS1

**Figure 4:**
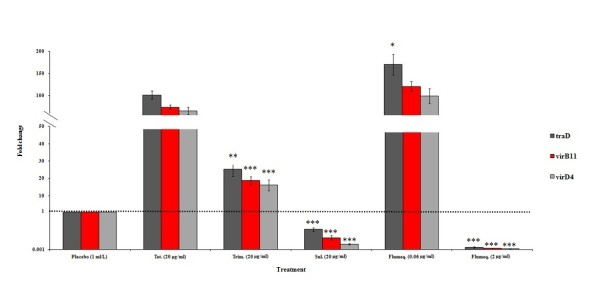
**Expression of three pRAS1 plasmid mobility genes in intestinal samples from adult zebrafish 48 h post treatment (72 h post experimental infection) relative to placebo treatment**. Error bars represent ± SEM (based on variation between 6 adults per treatment group). Differences were considered significant at (*) *p *< 0.05, (**) *p *< 0.01 and (***) *p *< 0.001 for mobility gene expression levels of tetracycline vs. other antibiotic treated zebrafish in each intestinal tissue analyzed.

The expression of *traD*, *virB11 *and *virD4 *was strongly induced by ineffective treatment (tetracycline, trimethoprim and sub-inhibitory levels of flumequine) and strongly reduced by treatment with effective concentrations of flumequine [Figure 4]. However, ineffective sulphonamide slightly reduced the expression of these genes.

### Immune responses following effective and ineffective treatments

**Figure 5:**
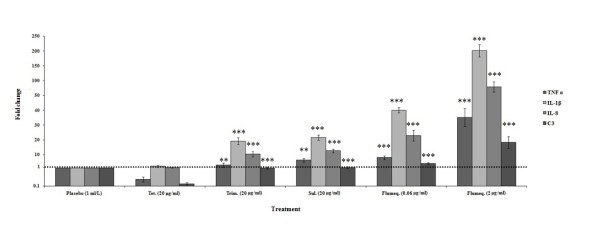
**Expression of selected inflammatory and immune response genes in the entire intestine of experimentally infected zebrafish 48 h post antibiotic treatment, relative to the expression in placebo treated fish (ref. Figure 2)**. Error bars represent ± SEM (based on variation between 6 adults per treatment group). Differences were considered significant at (*) *p *< 0.05, (**) *p *< 0.01 and (***) *p *< 0.001 for immune response levels of tetracycline vs. other antibiotic treated zebrafish in each intestinal tissue analyzed.

Our results revealed a strong up-regulation of all four analyzed immune related genes after effective flumequine treatment. An induction of some of these genes was observed even after ineffective treatment with trimethoprim, sulphonamide and a sub-lethal level of flumequine, whereas ineffective tetracycline treatment apparently suppressed two of the innate immune response mediators [Figure 5].

## Discussion

In this study, we have for the first time employed an experimental zebrafish infection- treatment model to mimic the conditions under which antibiotic resistance (mediated by a naturally occurring R-plasmid) transfer takes place in the intestinal microbiota during an infection caused by a resistant pathogen treated with effective or ineffective antibiotic treatments.

We were able to establish an infection with *A. hydrophila *resulting in disease symptoms similar to those previously described [10,11] but with no mortality 3 days post- infection, as intended in our study design. Rodriguez et al. [10] and Pullium et al. [11] observed per-acute cases of *A. hydrophila *infection with high mortality rates within a few hours possibly related to intraperitoneal injection of bacterial extracellular toxins and/or enzymes. In our study, re-isolation of the challenge organism from the kidneys of exposed fish supported the clinical findings and confirmed systemic infection. It has also been shown that *A. hydrophila *produces an array of virulence factors that induce strong inflammatory responses [34-36]. The induction kinetics of some of the zebrafish intestinal immune system genes revealed an Acute Phase Response (APR), that is the immediate host inflammatory reaction which counteract challenges such as tissue injury and infection [37]. In the current study *A. hydrophila *infection resulted in a clear increase in expression of the genes encoding the pro-inflammatory cytokines TNF α, IL-1β and IL-8. These cytokines are important inducers of APR resulting in increased production of Acute Phase Proteins (APPs) [38], such as C3. C3 is central in elimination of bacterial threats [39]. A systematic study of APR in zebrafish has shown striking similarities with mammals in function and induction of involved genes [25]. The fact that 1 IL-1β and IL-8 are highly induced while C3 remains moderately expressed is consistent with the expected expression profile at the early stages of infection (3 days in our case).

The composition of the zebrafish intestinal bacterial microbiota and its interaction with the host and the environment has previously been studied by cultivation and culture-independent methods [28,40]. In the present study this microflora and the experimentally introduced pRAS1 harboring *A. hydrophila *were impacted by various antibiotic treatments. Recent studies have shown that Real-Time PCR with species-specific or universal probes is an accurate and sensitive method for quantification of total bacterial populations as well as individual species from the intestinal contents [41-45].

In our study a broad spectrum of 16S rDNA primers were used since bacteria can have different genome sizes and different *rrn *operon copy numbers. There are different concepts for considering the *rrn *operon numbers in quantitative 16S rDNA-based experimental systems [43,44,46]. Ott et al. [47], have provided accurate and stable figures of similar bacterial concentrations in clinical samples with application of universal primers and specific probes. In the present study, 16S rDNA gene copy numbers were significantly decreased after effective flumequine treatment, whereas sub-lethal flumequine or the clinically relevant ineffective tetracycline, trimethoprim and sulphonamide treatments caused minimal change. The reduction in 16S rDNA gene copy number following treatment with flumequine might be the result of killing of pathogenic *A. hydrophila *and a disturbed and reduced commensal flora. In mammals and humans, it is well known that antibiotics can change the composition of the bacterial populations in the intestines [48-50]. Studies concerning the distribution of antibiotic resistant bacterial isolates in zebrafish facilities are, however, limited. Previous studies performed in our laboratory Cantas et al. [28], have shown a relatively low level of tetracycline (12-20%), trimethoprim (25-32%), sulphonamide (28-36%) and quinolone (0.5-4.8%) antibiotic resistant bacteria in the Gram negative cultivable gut flora in four different zebrafish facilities, one of which supplied the zebrafish for the present study. This would leave potential recipient flora for plasmid transfer in all treatment groups.

The minimal change in total 16S rDNA copy number following treatment with clinically relevant levels of tetracycline, trimethoprim and sulphonamide may be explained by multiplication of the resistant *A. hydrophila *pathogen due to the decreased competition following killing of the susceptible part of the normal intestinal microbiota.

The active involvement of the selected *tra*-genes in the DNA conjugation process is described [18]. The *traD *gene encodes an inner membrane protein with putative ATPase activity for DNA transport during bacterial conjugation. This protein forms a ring-shaped structure in the inner membrane through which DNA is passed to the transferosome [18,51]. However, it has been shown that the *virB4 *and *virD11 *genes may, in addition, mediate conjugative transfer via a C-terminal ATPase function during pili assembly which is more efficient on surfaces than in liquids [52,53]. pRAS1 is transferred approximately 1000× faster on solid surfaces compared to the frequency in liquid media [Kruse and Sørum 1994, unpublished data]

The genes of the conjugative transfer system studied i.e. *traD, virB11 *and *virD4*, were found to be differently expressed between the treatment groups. The expression of transfer genes was found to be low following sulphonamide and flumequine treatment, whereas treatment with a sub-inhibitory level of flumequine, clinical relevant levels of tetracycline and trimethoprim resulted in increased expression. Several factors have been proposed that could explain these differences; i) the susceptible gut microbiota was reduced in number leaving behind a variable number of potential conjugation recipients [54], ii) the donor potential and the genetic advantages/disadvantages of the specific plasmid in conjugating to the available recipient population [55], iii) the antibiotic itself might regulate the higher or lower expression levels of pRAS1 mobility genes resulting in possible different transfer frequencies. An increased transfer frequency induced by antibiotic exposures (tetracycline and trimethoprim) has been demonstrated for conjugal transfer of pRAS1 plasmid in sediment microcosm experiments [56].

A most remarkable result of the current study was the strongly increased expression levels of the selected plasmid transfer genes in the intestinal microbiota following treatment with tetracycline, trimethoprim (plasmid encoded resistance) and ineffective concentrations of flumequine. The low concentration of the quinolone flumequine was chosen to mimic the low concentration in the intestinal lumen when administering the drug intramuscularly or intravenously for treatment purposes, in in-appetent animals offered in-feed antibiotics, or by exposure to environmental residues from the water [55,57,58]. It has been shown that administration of sub-therapeutic levels can interfere with DNA replication (e.g. quinolones) [59,60], folic acid synthesis (e.g. trimethoprim) [61], protein synthesis (e.g. tetracycline) [62] as well as cell wall synthesis (e.g. β-lactams) [63] and may induce the so-called SOS response [64] which can promote acquisition and dissemination of antibiotic resistance genes [57,65]. Thus, our results reinforce the need for great caution in the use of SOS-inducing antibiotics to avoid induction of resistance transfer following antibiotic therapy. It is known that the LexA protein as part of the SOS response binds to the LexA box preceding the *intI *gene and thereby increasing the transcription rate of the *intI *gene resulting in an increased gene cassette exchange rate in the integron [66]. There is no recognized LexA box found close to the promoters of the *traD*, *virB11 *and *virD4 *genes of the pRAS1 plasmid sequence (data not shown). However, the occurrence of LexA targets in promoter sequence areas *in vivo *without the existence of a putative LexA box in the DNA sequence has been demonstrated. This indicates the assistance by an additional unknown factor in regulation of LexA gene expression *in vivo *[67].

An equally remarkable finding was the impact of antibiotic treatments on the expression of innate immunity genes. The decreased TNF α and C3 expression in the zebrafish's intestine after non-effective tetracycline treatment is in accordance with earlier reports [68,69] relating tetracyclines to posttranscriptional blockage of cytokine production [70]. Whereas, sulphonamide and trimethoprim treatments that have no impact on the growth of pathogenic *A. hydrophila *had little impact on IL-1β and IL-8, as expected. In contrast, the sub-inhibitory level of flumequine caused 40 and 20 fold increases in the expressions of IL-1β and IL-8, respectively. In addition effective flumequine treatment caused 200 and 100 times higher expressions of those genes, respectively. Hypothetically, this may be related to the immunomodulatory properties of those drugs [71,72] and in the diminished number (killed) of pathogenic *A. hydrophila *that can no longer depress the immune system by its virulence factors when the effective flumequine treatment was employed [73,74].

We have for the first time termed this clear, aggressive, immunological activity at the molecular level as 'Charged Immune Attack, (CIA)', which describes the inevitably strong revenge of the innate immune response against the weakened bacterial infection, as mediated by a short period with an effective antimicrobial treatment. The reason for this bias is not known, but both human and veterinary medical practitioners have observed that a single dose of antibiotics, sometimes surprisingly, may cure an infection. We think that the current results provide a glance into subtle and immediate effects of chemotherapy on the host's innate immune system that may be responsible for such outcomes. Further studies are needed to shed new light on the current findings and to clarify the underlying mechanisms.

For methodological reasons, most studies of *in vivo *conjugal plasmid transfer have been performed by adding donors and limited numbers of recipients in germ free animals [75,76] or by challenging conventional fish with genetically tagged bacteria [77]. To the best of our knowledge, this is the first report on the effect of antibiotic treatment of an infection on the expression of the *tra *genes of an R-plasmid harbored by the infecting pathogen and the early immune signals in a host model. Real-Time PCR technology offers a fast and reliable quantification of the mRNA production of any target sequence in a sample [78]. The results add information to our knowledge about development of antibiotic resistance in infected hosts including the clinical infection treatment and control scenario.

## Conclusions

As expected the control of the *A. hydrophila *infection of zebrafish failed when tetracycline, trimethoprim and sulphonamide were used due to the R-plasmid (pRAS1) harbored by the pathogen. The same result was identified as expected when sub-inhibitory levels of flumequine were employed, whereas an effective dosage of flumequine reduced the clinical symptoms and controlled the pathogen and transfer of pRAS1. At the same time, the ineffective therapeutants tetracycline, trimethoprim and sub-inhibitory concentrations of flumequine increased the expression levels of plasmid mobility genes. The results should be taken into account by physicians and veterinarians when prescribing antibiotic drugs, underscoring the need to avoid risk for augmenting the transfer of genetic drug resistance elements to commensal microbiota.

This is the first combined *in vivo *study of antibiotic treatment on the innate immune system of the host and the conjugative activity of an R plasmid. A particularly valuable observation relates to the increased activity of the innate immune system caused by antibiotic exposure, even with ineffective drugs (R-plasmids) and at sub-therapeutic levels.

## Authors' contributions

LC conceived the idea for the study, formulated the research hypothesis, designed the experiment, performed the fish infection studies, performed the sampling and data collection, carried out all bacteriological laboratory work including the quantitative Real-Time PCR tests, performed the statistical analysis and interpretation of the data, formulated the underlying causes and drafted the manuscript. PJM contributed to the study design and *in vivo *protocol, and supervised the zebrafish experimental infection trial. HS contributed to acquisition of funds, provided guidance to the formulation of the underlying hypothesis, supervision of the laboratory work and the interpretation of the data. All authors discussed the results, revised and adopted the manuscript.
